# Relationship between age and remimazolam dose required for inducing loss of consciousness in older surgical patients

**DOI:** 10.3389/fmed.2024.1331103

**Published:** 2024-04-29

**Authors:** Jin-Chao Song, Xiao-xi Wang, Xiang Fu, Wei Chen, Shu-heng Tang, Fang Deng, Hua Yang, Wen Liu

**Affiliations:** ^1^Department of Anesthesiology, Shidong Hospital Affiliated to University of Shanghai for Science and Technology, Shanghai, China; ^2^Department of Anesthesiology, Eastern Hepatobillary Surgical Hospital, Naval Medical University, Shanghai, China; ^3^Department of Pharmacy, Shidong Hospital Affiliated to University of Shanghai for Science and Technology, Shanghai, China

**Keywords:** older surgical patients, remimazolam, loss of consciousness, anaesthetics, intravenous anesthesia

## Abstract

**Background:**

Remimazolam is a new ultra-short-acting benzodiazepine for procedural sedation and general anaesthesia, characterised by rapid onset of action, quick recovery, and organ-independent metabolism. Older patients tend to sustain more treatment-emergent adverse events (TEAEs) and worse perioperative prognoses after receiving remimazolam. However, few studies have investigated the appropriate dose of remimazolam for loss of consciousness (LOC) in geriatric patients. We designed this study to provide evidence for dose references and elucidate the relationship between age and remimazolam requirement for inducing LOC during anaesthesia induction.

**Methods:**

Exactly 120 patients scheduled for general surgery under general anaesthesia were included and divided into two groups: Group A (60 patients, 18–64 years) and Group B (60 patients, ≥ 65 years). LOC, defined as a Modified Observer’s Assessment of Alertness and Sedation score at 1 had been reached, emerged after all participants received a continuous infusion of remimazolam at a rate of 0.05 mg/kg/min.

**Results:**

The remimazolam required for inducing LOC was 0.26 and 0.19 mg/kg in groups A and B, respectively, and the remimazolam dose in group B decreased by 26.9% compared to group A. According to the bivariate linear correlation analysis, remimazolam requirement was negatively correlated with age. Multivariable linear regression models and further adjustments for potential impact factors indicated that age was an independent factor for the remimazolam dose required for LOC.

**Conclusion:**

This study demonstrated that age was significantly and independently correlated with the remimazolam requirement for inducing LOC. To obtain haemodynamic stability during the induction of general anaesthesia, appropriately reducing the remimazolam dose is recommended for geriatric patients.

## Highlights

- Age was significantly and independently correlated with the dose of remimazolam required for inducing loss of consciousness (LOC).
- Appropriately reducing the remimazolam dose is recommended for geriatric patients to obtain haemodynamic stability during the induction of general anaesthesia.

## 1 Introduction

Remimazolam, acting on gamma-aminobutyric acid (GABA) receptors, is a new ultra-short-acting benzodiazepine for intravenous sedation and anaesthesia, characterised by rapid onset of action, quick recovery, and organ-independent metabolism (1). Additionally, its sedative and hypnotic effects can be reversed by flumazenil, similar to other benzodiazepines (2), Since older patients are more vulnerable to cardiorespiratory inhibitory effects and have longer drug metabolism time (3), administration of remimazolam presents a high safety profile, producing less effect on circulatory and pulmonary functions and fewer other TEAEs (4). Hypotension is less frequent when patients receive remimazolam compared to propofol and midazolam (5, 6). TEAEs such as administration site pain, increased bilirubin, and reduced respiratory rate are also less common, particularly compared with propofol (7). Furthermore, remimazolam offers rapid recovery and early restoration of cognitive function, reducing the risk of postoperative cognitive impairment commonly occurring in geriatric patients after major surgeries (8). A recent study indicated that remimazolam administration for anaesthetic induction had an inhibitory effect on postoperative cognitive dysfunction compared with propofol (9).

As an anaesthetic for adult patients, the optimal doses of remimazolam for induction and maintenance are 6 or 12 mg/kg/h and 1 mg/kg/h, respectively (6) and it is safe and effective to recommend a loading dose of 12 mg for sedation and reducing anaesthetic induction time (10). However, the appropriate doses of remimazolam for older patients are yet to be evaluated because of their hypersensitivity to sedative drugs and degeneration of cardiorespiratory function. Hence, we designed this study to investigate the effect of age on remimazolam dose requirement for inducing LOC in geriatric surgical patients.

## 2 Patients and methods

### 2.1 Participants

This study was approved by the Institutional Research Ethics Committee of Shidong Hospital. The registration number of this clinical trials is ChiCTR2300069582.^1^ Patients aged 18 years and older with American Society of Anesthesiologists (ASA) physical status I to III and body mass index (BMI) between 18 and 32 kg/m^2^ scheduled for general surgery or orthopedic surgery under general anaesthesia in Shidong Hospital were considered eligible. All the patients in the study were Chinese, Asian and yellow.

Exclusion criteria included: patients who were allergic to the planned medication at the time of enrolment, who were known to have severe cardiac and cerebral vascular diseases, central nervous system diseases, haematologic and metabolic diseases, increased intracranial pressure, and hepatic or renal abnormalities. Patients with a history of alcohol or drug abuse and use of analgesics or neuromodulating medications were also excluded. Written informed consent was obtained from all the participants.

### 2.2 Study protocol

In total, 120 patients who met the inclusion and exclusion criteria from March to August 2022 were enrolled and divided into two groups according to age: Group A (60 patients, 18–64 years) and Group B (60 patients, ≥ 65 years). A 6 h absolute diet regimen and no premedication were ensured for all patients before the induction of anaesthesia. After being transferred to the operating room, the following parameters were monitored: non-invasive blood pressure, heart rate (HR), electrocardiogram, pulse oxygen saturation (SpO_2_), and end-tidal carbon dioxide. Ringer’s lactate solution was administered at 16–18 mL/kg/h during preoxygenation. LOC, defined as the Modified Observer’s Assessment of Alertness and Sedation score at 1 had been reached, emerged after continuous infusion of remimazolam tosilate (HengRui Medicine Co., Ltd., Jiangsu, China) at 0.1 mg/kg/min. An anaesthesiologist blinded to this study assessed the progress of induction and determined a successful induction once a Modified Observer’s Assessment of Alertness and Sedation score at 1, after which sufentanil (Yichang Humanwell Pharmaceutical Co., Ltd., Yichang, China) 0.3–0.5 μg/kg and cisatracurium (Nanjing Jianyou Biochemical Pharmaceutical Co., Ltd., Nanjing, China) 0.15–0.2 mg/kg were administered intravenously, and tracheal intubation was performed 3 min later. General anaesthesia was maintained with remifentanil (0.2–0.3) μg/kg/min and sevoflurane (1.2–2.5%).

The ventilatory frequency was adjusted to maintain the end-tidal carbon dioxide concentration at 35–45 mmHg. Ephedrine (5 mg) was administered intravenously when the patient’s systolic blood pressure (SBP) was below 90 mmHg or 30% lower than the basic blood pressure, and 0.5 mg atropine was administered when the patient’s heart rate was below 50 bpm. Anaesthesiologists exerted positive pressure-assisted ventilation with a non-invasive face mask when the patient’s pulse oxygen saturation was below 92%.

We detected several vital signs in this study, including mean arterial pressure (MAP), HR, and SpO_2_ at six different points: T0, before remimazolam administration; T1, LOC; T2, 3 min after the administration of analgesic and muscle relaxant; T3, the moment after intubation; T4, 5 min after intubation; and T5, 10 min after intubation. The doses of remimazolam required to induce LOC; ejection fraction (EF); international normalised ratio (INR); and albumin (ALB), bilirubin, alanine aminotransferase (ALT), aspartate aminotransferase (AST), serum creatinine (Scr), and blood urea nitrogen (BUN) levels were collected and recorded, as well as adverse events such as hypotension, bradycardia, and hypoxaemia.

### 2.3 Statistical analysis

The primary goal of this study was to compare remimazolam dose requirements for LOC in geriatric surgical patients. Group sample size was calculated based on differences in remimazolam requirements for LOC in a pilot study, in which the mean remimazolam requirement was 0.25 ± 0.13 (*n* = 10) mg/kg in the adult group and 0.20 ± 0.06 (*n* = 10) mg/kg in the elderly group. Exactly 58 samples for each group met the requirement of *α* = 0.05 and power = 0.8, using the formula: *n* = 15.7/ES^2^ + 1, where ES = effect size = (difference between groups) / (mean of the standard deviation between groups).

Normally distributed continuous variables are presented as the mean (standard deviation, SD), skewed distributed continuous variables as the median (interquartile range, IQR), and categorical variables as frequency (percentage). Student’s *t*-test or Mann–Whitney U test were used to analyse patients’ baseline characteristics and haemodynamic changes, as appropriate. Pearson chi-square test was applied to categorical variables. Pearson’s or Spearman’s correlation coefficients were used to assess the correlations between the remimazolam dose requirement and other parameters according to whether the normal distribution assumption of data was satisfied or not.

Three multivariable linear regression models were established to determine whether age was independently correlated with remimazolam dose: Model I: unadjusted; Model II: adjusted for sex and BMI; and Model III: further adjusted for ALB, ALT, Scr, and BUN levels. Variables in the model were selected to determine whether they were potential impact factors if the univariate analysis result was at *p*-value <0.05. We also included several indices highly relevant to remimazolam drug metabolism and clinical liver and kidney function (11). A collinearity diagnosis was performed to avoid highly interrelated variables in the model. We conducted smooth curve fitting to address the linearity between age and remimazolam dose after adjusting for potential effect variables. All statistical analyses were performed using IBM SPSS Statistics version 26 and R Project for Statistical Computing version 4.2.2. Statistical significance was considered when *p* value was <0.05.

### 2.4 Sensitivity analysis

We used R^2^ to identify the relationship between age and remimazolam dose and further adjusted for confounding factors, which were selected based on clinical expertise and previous studies (11). Model I: unadjusted; Model II: adjusted for sex and BMI; and Model III: further adjusted for ALB, ALT, Scr, and BUN.

## 3 Results

**Figure 1:**
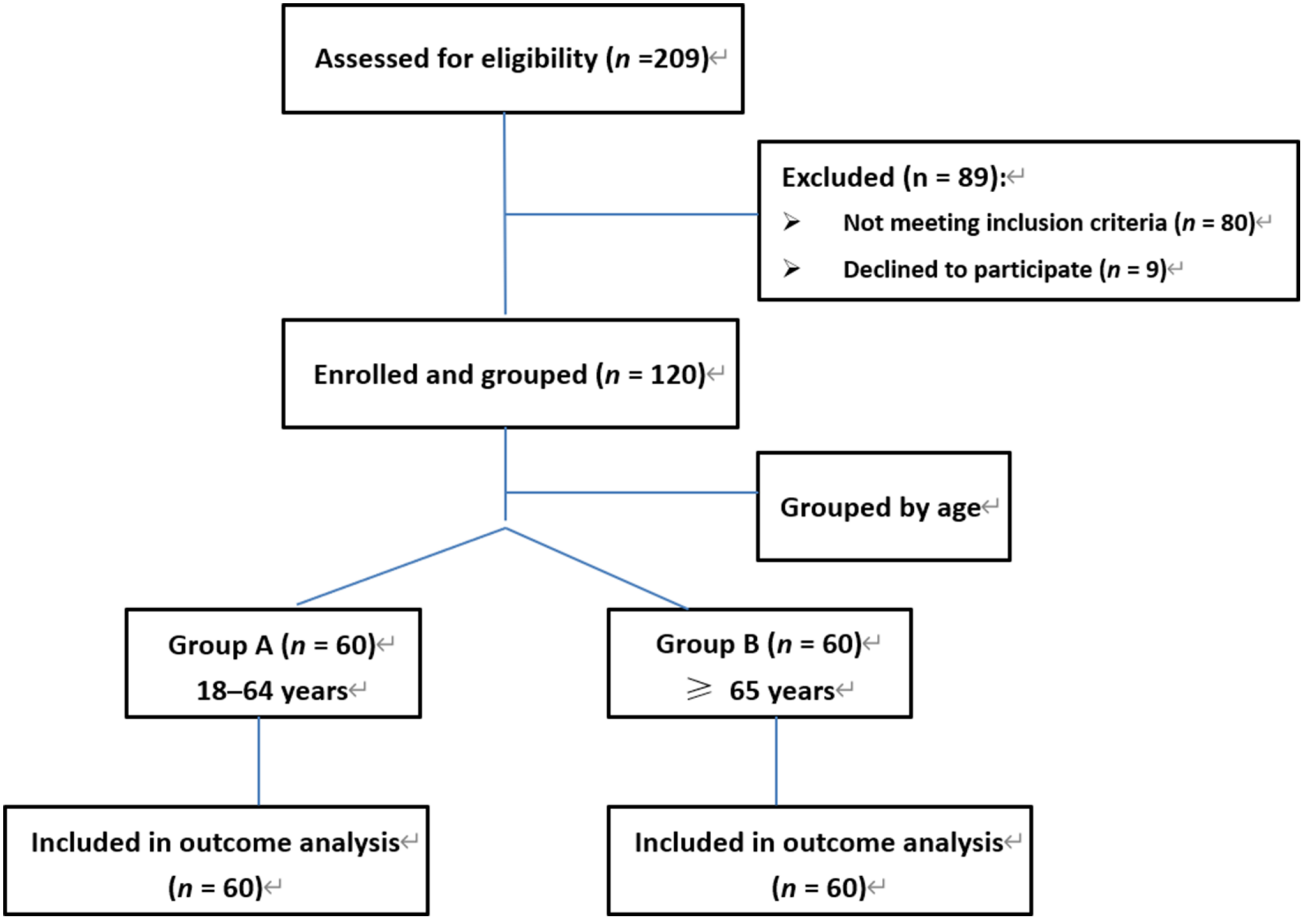
The study flow diagram.

Figure 1 shows the study flow diagram. The baseline characteristics of 120 patients, equally divided into groups A (53.5 ± 9.4, *n* = 60) and B (75.2 ± 7.0, *n* = 60), are presented in Table 1. No differences were observed in sex, BMI, bilirubin, ALT, and AST levels between the two age groups. However, patients in group B had significantly higher Scr levels (*z* = −2.1, *p* = 0.034), BUN levels (*z* = −2.5, *p* = 0.013), and INR (*z* = −2.8, *p* = 0.004) than those in group A, whereas EF (*z* = −3.5, *p* < 0.001) and ALB levels (*z* = −3.5, *p* = 0.011) were lower.

**Table 1:** Patient baseline characteristics.

	Group A	Group B	*p* value
	(*n* = 60)	(*n* = 60)	
Male, No. (%)	21 (35)	29 (48)	0.139
BMI, mean (SD), kg/m^2^	23.65 (3.18)	23.68 (2.71)	0.953
EF, median (IQR), (%)	64.00 (63.00–65.00)	63.00 (62.00–64.00)	<0.001^***^
INR, median (IQR)	0.93 (0.90–0.98)	0.97 (0.92–1.01)	0.004^**^
Liver function			
Albumin, median (IQR), g/L	41.40 (39.33–43.85)	38.85 (35.63–44.03)	0.011^*^
Bilirubin, median (IQR), μmol/L	11.50 (8.65–15.83)	13.30 (10.00–16.68)	0.225
ALT, median (IQR), U/L	19.50 (13.00–25.75)	17.00 (13.00–27.50)	0.445
AST, median (IQR), U/L	21.50 (17.00–26.75)	22.00 (19.00–26.00)	0.595
Kidney function			
Scr, median (IQR), μmol/L	61.40 (52.73–73.13)	67.40 (57.18–82.85)	0.034^*^
BUN, median (IQR), mmol/L	4.92 (3.91–6.23)	5.93 (4.50–7.70)	0.013^*^
ASA physic status, n (%)			0.063
I	9 (15.0)	6 (10.0)	
II	42 (70.0)	34 (56.7)	
III	9 (15.0)	20 (33.3)	
Operation time, mean (SD), min	76 (55)	103 (93)	0.056
Hospital stay post surgery, mean (SD), day	5.3 (3.9)	7.2 (5.5)	0.036^*^
Procedures, *n*			
General surgery	32	33	
Orthopedic surgery	3	11	
Gynecological surgery	17	7	
Urology surgery	8	9	

BMI, body mass index; EF, ejection fraction; INR, international normalized ratio; ALB, albumin; ALT, alanine aminotransferase; AST, aspartate aminotransferase; Scr, serum creatinine, BUN, blood urea nitrogen. *p* value in Student’s *t* test, Mann Whitney U test or Pearson chi-square test. ^*^p < 0.05, ^**^p < 0.01, ^***^p < 0.001.

**Figure 2:**
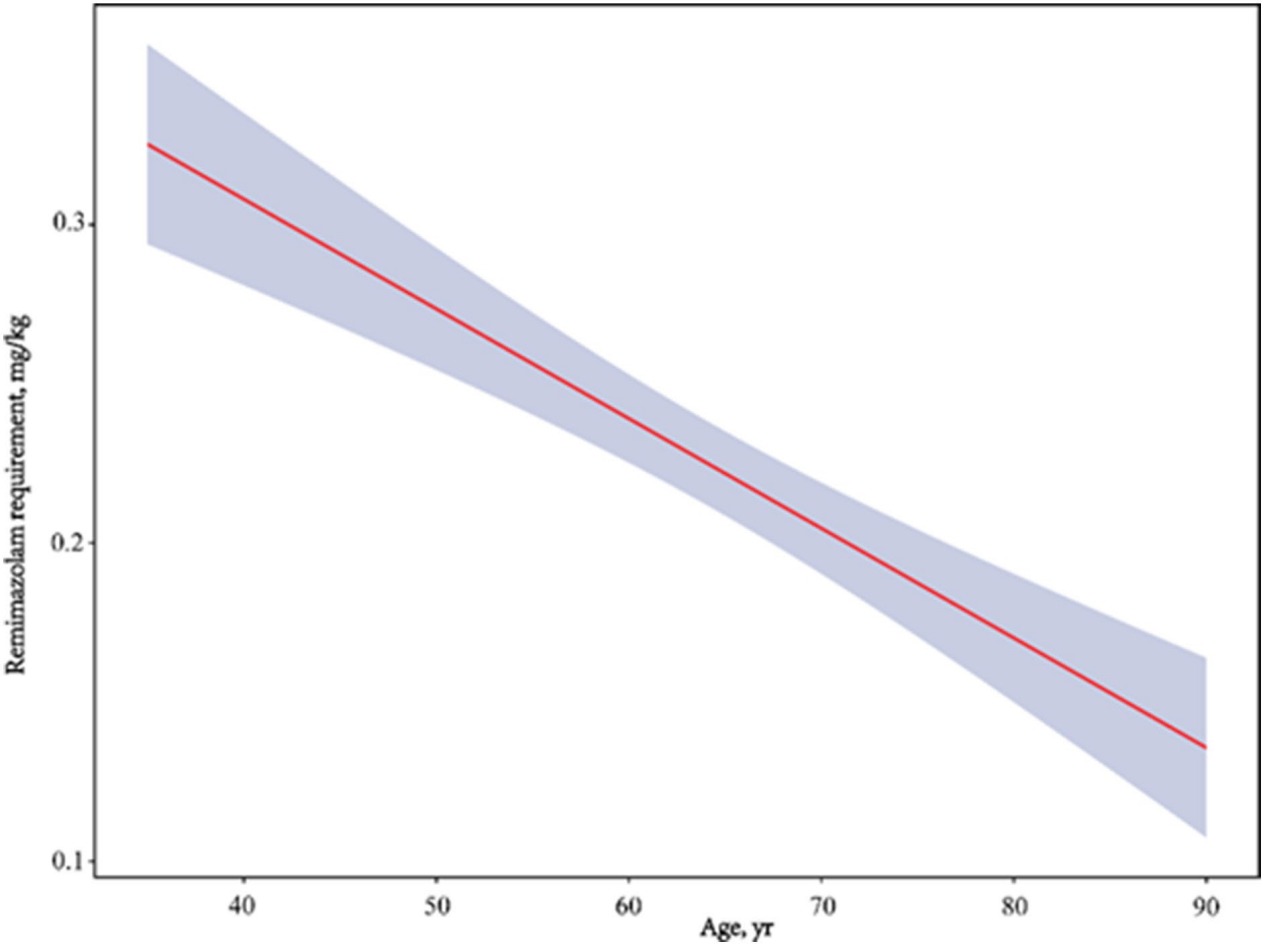
Adjusted dose–response linear between age and remimazolam requirement. Adjusted for gender, BMI, ALB, ALT, Scr and BUN.

A comparison of the remimazolam dose requirement for inducing LOC between groups A (0.26 ± 0.10 mg/kg) and B (0.19 ± 0.05 mg/kg) is shown in Table 2. We showed that the remimazolam dose for inducing LOC was significantly associated with age (*t* = 4.5, *p* < 0.001). Further, the remimazolam dose in group B decreased by 26.9% compared with that in group A. According to the bivariate linear correlation analysis in Table 3, we also found a significant negative correlation between remimazolam requirement and age (*r* = −0.543, 95% confidence interval (CI): −0.650 to-0.422, *p* < 0.001); with a significant decline in BMI, remimazolam requirement offered a corresponding increase (*r* = −0.265, 95% CI: −0.478 to-0.031, *p* = 0.003). In contrast, remimazolam dose requirement was positively correlated with ALB levels, which was statistically significant (*r* = 0.252, 95% CI: 0.052–0.414, *p* = 0.005). Other organic function indicators were not significantly correlated with the remimazolam dose required. Furthermore, we used smooth curve fitting to identify the linear relationship between age and remimazolam dose required after adjusting for sex, BMI, ALB, ALT, Scr, and BUN, which were statistically significant (Figure 2).

**Table 2:** Remimazolam requirement for LOC in each group.

	Group A (*n* = 60)	Group B (*n* = 60)	*p* value
Remimazolam dose mean (SD), mg/kg	0.26 (0.10)	0.19 (0.05)	<0.001^***^

*p* value in Student’s *t* test. ^*^p < 0.05, ^**^p < 0.01, ^***^p < 0.001.

**Table 3:** Correlation coefficients between age and remimazolam requirement.

	Remimazolam requirement, Mg/kg		
	r	95% CI	*p* value
Age, yr	−0.543	−0.650 ~ −0.422	<0.001^***^
BMI, kg/m^2^	−0.265	−0.478 ~ −0.031	0.003^**^

	Remimazolam requirement, Mg/kg		
	r_s_	95% CI	*p* value
EF, %	0.036	−0.148 ~ 0.241	0.698
INR	0.019	−0.154 ~ 0.218	0.837
Albumin, g/L	0.252	0.052 ~ 0.414	0.005^**^
Bilirubin, μmol/L	−0.106	−0.300 ~ 0.072	0.249
ALT, U/L	−0.117	−0.298 ~ 0.061	0.203
AST, U/L	−0.091	−0.266 ~ 0.088	0.323
Scr, μmol/L	−0.166	−0.325 ~ 0.006	0.069
BUN, mmol/L	−0.159	−0.326 ~ 0.017	0.082

*p* value in Pearson’s (r) and Spearman’s (r_s_) correlation coefficients. ^*^p < 0.05, ^**^p < 0.01, ^***^p < 0.001.

Multivariable linear regression models were used to confirm the independent role of age in remimazolam requirement. They were further adjusted by three different models composed of potential confounding factors. Model I served as an unadjusted model that solely integrated the age variable, aiming to preliminarily elucidate the influence of age on remimazolam dosage. Following the outcomes of univariate linear regression analyses, BMI and sex were deemed significant (*p*-value <0.05) and consequently included in Model II. Subsequent to these findings, in alignment with clinical practices and research outcomes (11), Model III incorporated ALB, ALT, Scr, and BUN. These variables were selected for their substantial relevance to clinical liver and kidney function, as well as their impact on remimazolam metabolism (Supplementary material S1). Collinearity diagnostics in multivariable linear regression models did not identify any highly interrelated variables that were excluded (Supplementary material S2). Our study demonstrated that an increase in the standard deviation (SD) of 1 year was associated with a reduction in the requirement of remimazolam by 0.048 mg/kg (95% CI: −0.062 to-0.035, *p* < 0.001) in Model I. Upon adjustment for potential confounding factors, we observed that a one-year SD increase corresponded to a decrease in remimazolam requirement by 0.045 mg/kg (95% CI: −0.059 to-0.032, *p* < 0.001) in Model II and a similar reduction of 0.045 mg/kg (95% CI: −0.060 to-0.031, p < 0.001) in Model III. Compared with young and middle-aged patients (Group A), aging patients (Group B) required less remimazolam to lose consciousness in the unadjusted model, which was statistically significant (*β* = −0.067, 95% CI: −0.096–-0.038, *p* < 0.001). After adjusting for BMI and sex in model II (*β* = −0.063, 95% CI: −0.091–-0.035, *p* < 0.001) and further adjusting for ALB, ALT, Scr, and BUN levels in model III (*β* = −0.058, 95% CI: −0.088–-0.027, *p* < 0.001), consistent and significant results were observed, respectively (Table 4).

**Table 4:** Effects of age on remimazolam requirement.

Age,yr	Model I		Model II		Model III	
	β (95% CI)	*p* value	β (95% CI)	*p* value	β (95% CI)	*p* value
Per 1 SD	−0.048 (−0.062–−0.035)	<0.001^***^	−0.045 (−0.059–−0.032)	<0.001^***^	−0.045 (−0.060−−0.031)	<0.001^***^
Group A	0.00 (references)		0.00 (references)		0.00 (references)	
Group B	−0.067 (−0.096–−0.038)	<0.001^***^	−0.063 (−0.091–−0.035)	<0.001^***^	−0.058 (−0.088–−0.027)	<0.001^***^

Model I, unadjusted; Model II, adjusted for gender and BMI; Model III, further adjusted for ALB, ALT, Scr and BUN. *p* value in multivariable linear regression models. ^*^p < 0.05, ^**^p < 0.01, ^***^p < 0.001.

**Figure 3:**
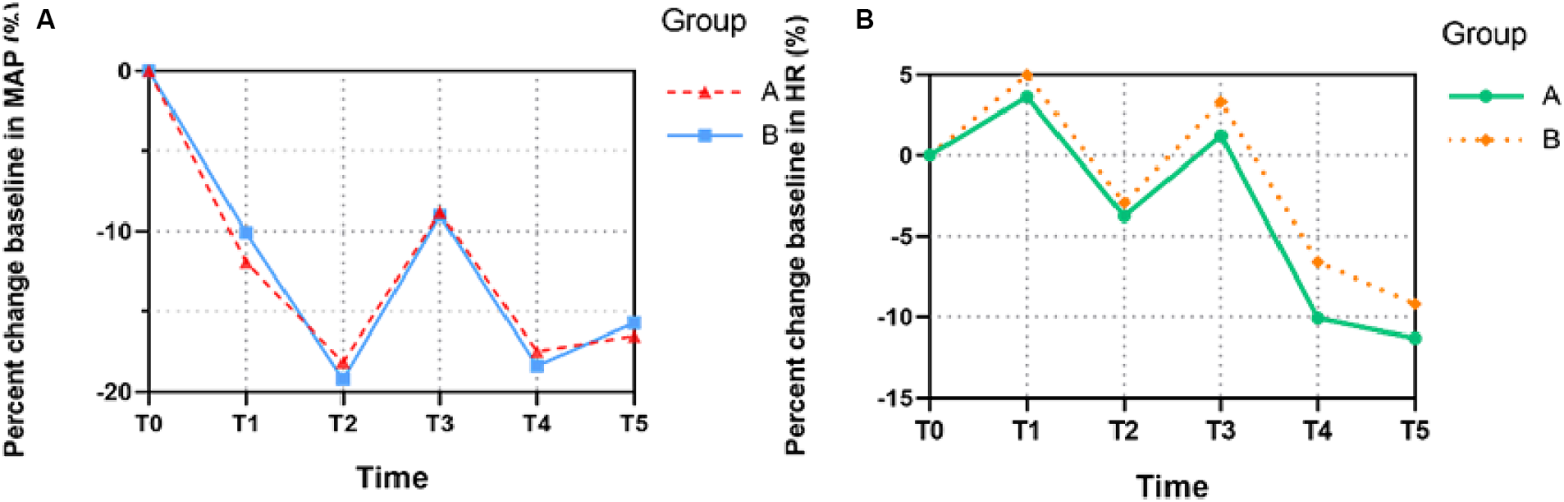
The trends of patients’ hemodynamic profiles relative to baseline during the induction of anesthesia. **(A)** Mean arterial pressure (MAP), **(B)** Heart rate (HR). The percent changes of MAP and HR at six different time points in each group were compared. All the values are presented as mean. T0, before remimazolam administration; T1, LOC; T2, 3 min after the administration of analgesic and muscle relaxant; T3, the moment after intubation; T4, 5 min after intubation; T5, 10 min after intubation.

In Figure 3, compared with the baseline, the MAP of both groups decreased to the lowest level at T2 (3 min after the administration of analgesics and muscle relaxants) and then exhibited stable profiles until T5 (10 min after intubation), which indicated no statistically significant difference between the groups (MAP: T1, *t* = −0.94, *p* = 0.351; T2, *t* = 0.51, *p* = 0.611; T3, *t* = 0.08, *p* = 0.938; T4, *t* = 0.34, *p* = 0.732; T5, *t* = 0.29, *p* = 0.771). Similarly, no statistically significant difference was found in the HR between each group, which reached relatively high levels at T1 (LOC) and T3 (the moment after intubation) and then gradually decreased (HR: T1, *t* = −0.63, *p* = 0.531; T2, *t* = −0.32, *p* = 0.752; T3, *t* = −0.74, *p* = 0.463; T4, *t* = −1.4, *p* = 0.161; T5, t = −0.77, *p* = 0.445).

## 4 Discussion

In this study, we investigated the appropriate dose of remimazolam to induce LOC during anaesthetic induction in older surgical patients. We found that the dose of remimazolam required for LOC significantly decreased with age, and age was considered to play an important and independent role in effective and safe induction. This provides further resources on the current limited data on optimal doses of remimazolam for inducing LOC, especially in older surgical patients.

In this study, we allocated patients into two groups based on age, showing that increasing age notably reduced the remimazolam dose required for LOC (Figure 2). We found that the remimazolam requirement of patients >65 years for LOC was 0.19 mg/kg, which is 23.7% lower than that reported for 60–79-year-old patients (0.249 mg/kg) in a previous study (12). Additionally, the proposed optimal doses of remimazolam for patients aged 60–80 and > 80 years by Chae et al. (13) were 0.19–0.25 and 0.14–0.19 mg/kg, respectively. Our findings are in accordance with the principle of minimal administration of remimazolam, as higher doses can be unnecessarily excessive in older patients.

The bivariate linear correlation analysis revealed that remimazolam requirement was positively correlated with ALB levels and negatively associated with BMI (Table 3). There was more than 91% plasma protein binding, predominantly to human serum albumin, and we believe that the unbound active fraction produces the anaesthetic effect (14). Therefore, the changes in ALB levels significantly influenced the optimal dose of remimazolam for inducing LOC. Although a larger initial dose of remimazolam may be required in overweight patients because of its ability to distribute into excess adipose tissue, our results showed an inverse relationship between remimazolam dose requirement and BMI. A reduced dose of remimazolam for LOC may be safe and effective in terms of the effect of being overweight on patients’ organic function and drug metabolism.

To determine whether patient age had an independent role in the remimazolam dose requirement for inducing LOC, several potential impact factors, such as sex, BMI, and ALB, ALT, Scr, and BUN levels, were included and further adjusted (Table 4). After performing multivariable linear regression models and further adjustments, we found that age was independently and significantly correlated with the remimazolam dose required. As a hypnotic agent with a stable haemodynamic profile, remimazolam was reported to have a lower incidence of cardiovascular and respiratory depression than propofol, especially in procedural sedation (7). However, aging strongly correlates with a series of organic degenerations and abnormal drug reactions involving lower cardiac output, decreased oxygenation, impaired hepatic and renal function, cognitive impairment, and hypersensitivity to anaesthetics (15). Aging alters the mechanical properties of the respiratory system, leading to a reduction in arterial oxyhaemoglobin saturation and impairment of the hypoxic response (16). Connective tissue stiffening and elastin damage are observed in older patients, contributing to higher blood pressure and lower stroke volume (17). Remimazolam has been reported to cause fewer TEAEs, such as perioperative hypotension, compared to propofol and midazolam (as described above) (5–7). However, a recent study reported that increasing remimazolam dose was accompanied by an increased incidence of hypotension, suggesting the need to reduce remimazolam doses during induction after considering confounding factors (18).

It is well known that postoperative cognitive impairment is among the most common clinical complications in geriatric patients and is highly relevant to surgical interventions at an advanced age (19). Currently, many anaesthetics administered in clinical practice act predominantly on GABA_A_ receptors, such as benzodiazepines and propofol. They induce sedation by increasing the opening frequency and permeability of the chloride ion channel of the nerve cell membrane (20). As a primary inhibitory neurotransmitter in the central nervous system, GABA may lead to cognitive impairment by disturbing cholinergic and glutamatergic neurones (21). More importantly, aging weakens the GABAergic system in both the quantity and function of neurones and synapses, which are involved in cognitive decline and neurological diseases in aged surgical patients. Liguz-Lecznar et al. discovered that decreased learning-dependent plasticity in the somatosensory cortex of aged mice could be associated with age-related delay in the GABAergic system (22). Meanwhile, GABA_A_ receptor α3 subunit fluctuations in the superior temporal gyrus were observed between young and old males, which indicated that dysregulation of GABA signalling in the human cortex was related to aging progression (23). Hence, remimazolam is recommended for rapid recovery and early restoration of cognitive function. In a study focused on hip replacement surgery, older patients who received remimazolam for general anaesthesia had significantly higher Mini-Mental State Examination scores at 1 and 3 days after surgery than those who received propofol (9). However, a recent clinical study showed that remimazolam may contribute to cognitive dysfunction in older patients after upper gastrointestinal endoscopy, suggesting that its administration for sedation or induction should be done cautiously, especially for geriatric patients (24).

There were five limitations in our study. First, the data from extremely aged and ASA-IV patients were insufficient, which might not provide enough evidence for the induction of patients at high risk. Second, we did not assess the postoperative cognitive function scores. Third, other factors possibly affecting the results, such as nutritional condition, were overlooked. Fourth, the assessor might know from the patient’s appearance which group the patient belongs to, but the assessor does not know the specific age of the patient. Fifth, we have not preformed a pharmacokinetic study, which is the gold standard for assessing drug pharmacokinetics.

In conclusion, this study demonstrated that age was significantly and independently correlated with the dose of remimazolam required for inducing LOC. To obtain haemodynamic stability during induction, an appropriate reduction of remimazolam dose is recommended for the geriatric population. Further studies are required to elucidate the perioperative prognostic obstacles with the administration of remimazolam, such as cognitive decline and hypotension.

## Data availability statement

The original contributions presented in the study are included in the article/Supplementary material, further inquiries can be directed to the corresponding authors.

## Ethics statement

The studies involving humans were approved by The Institutional Research Ethics Committee of Shidong Hospital. The studies were conducted in accordance with the local legislation and institutional requirements. The participants provided their written informed consent to participate in this study.

## Author contributions

J-CS: Conceptualization, Funding acquisition, Writing – original draft. X-xW: Formal analysis, Software, Writing – original draft. XF: Data curation, Visualization, Writing – original draft. WC: Data curation, Methodology, Writing – original draft. S-hT: Data curation, Resources, Writing – original draft. FD: Data curation, Methodology, Writing – original draft. HY: Conceptualization, Supervision, Writing – review & editing. WL: Conceptualization, Supervision, Writing – review & editing.

## Footnotes

^1^ http://www.chictr.org/cn/
