# Supplementary material for: Relationship between age and remimazolam dose required for inducing loss of consciousness in older surgical patients
Source: Front Med (Lausanne). 2024 Apr 29;11:1331103. doi: 10.3389/fmed.2024.1331103 (PMC11089116; doi:10.3389/fmed.2024.1331103)
Supplement: Supplementary file 1 [file Table_1.docx]

**Supplemental material**

**Supplemental material 1**

| **S1〡**Univariate analysis between remimazolam dose and each independent variable | | | |  |
| --- | --- | --- | --- | --- |
|  | **β** | **95% CI** | ***P* Value** |  |
| Age  BMI, kg/m^2^  EF%  INR  Albumin, g/L  Bilirubin,μmol/L  ALT, U/L  AST, U/L  Scr, μmol/L  BUN, mmol/L | -0.003  -0.008  0.001  0.170  0.003  -0.001  -0.001  -0.001  -0.001  -0.002 | -0.004~-0.002  -0.013~-0.003  -0.007~0.008  -0.035~0.375  0.000~0.006  -0.003~0.001  -0.002~0.000  -0.002~0.000  -0.001~0.000  -0.004~0.001 | <0.001^***^  0.003^**^  0.870  0.103  0.067  0.319  0.108  0.181  0.149  0.139 |  |
| *p* value in linear regression model. *p* <0.05, *; *p* <0.01, **; *p* <0.001, ***. | | | |  |
|  | | | | |
