# Supplementary material for: Relationship between age and remimazolam dose required for inducing loss of consciousness in older surgical patients
Source: Front Med (Lausanne). 2024 Apr 29;11:1331103. doi: 10.3389/fmed.2024.1331103 (PMC11089116; doi:10.3389/fmed.2024.1331103)
Supplement: Supplementary file 2 [file Table_2.docx]

**Supplemental material**

**Supplemental material 2**

| **S2〡**Collinearity diagnosis of variables in multivariable linear regression models | | | | | | | | | | | |
| --- | --- | --- | --- | --- | --- | --- | --- | --- | --- | --- | --- |
|  | **Model Ⅰ** | | |  | **Model Ⅱ** | | |  | **Model Ⅲ** | | |
|  | **Variables** | **VIF** | |  | **Variables** | **VIF** | |  | **Variables** | **VIF** | |
|  | age | | 1.000 |  | age | | 1.054 |  | age | | 1.255 |
|  |  | |  |  | gender | | 1.050 |  | gender | | 1.445 |
|  |  | |  |  | BMI | | 1.011 |  | BMI  ALB  ALT  Scr  Bun | | 1.075  1.228  1.128  1.396  1.113 |
| Model I: unadjusted; Model II: adjusted for gender and BMI; Model III: further adjusted for ALB, ALT, Scr and BUN. | | | | | | | | | | | |
